# Head and Neck Classic Hodgkin, T and NK Lymphomas with Eosinophilia

**DOI:** 10.1007/s12105-025-01751-9

**Published:** 2025-01-28

**Authors:** David T Danielson, Nadine S Aguilera, Aaron Auerbach

**Affiliations:** 1 Department of Pathology, Walter Reed National Military Medical Center, Bethesda, MD USA; 2 University of Virginia Health System, Charlottesville, VA USA; 3 Joint Pathology Center, Silver Spring, MD USA

**Keywords:** Eosinophilia, Hematopathology, Head and neck Pathology, Lymphoma, Leukemia

## Abstract

Eosinophilia is a notable feature in various hematological malignancies, including specific types of leukemias and lymphomas that may occur in the head and neck. In hematologic malignancies, eosinophilia can be primary, driven by genetic abnormalities, or secondary, resulting from cytokine and chemokine production by the neoplastic cells or the tumor microenvironment. This review examines the association between eosinophilia and head and neck hematolymphoid malignancies including Classic Hodgkin lymphoma, T-cell lymphoblastic leukemia, mature T and NK-cell lymphomas, and Langerhans cell histiocytosis. It explores the underlying mechanisms of eosinophilia in these malignancies, highlighting the role of chemokines and cytokines such as IL-5, TARC, and eotaxin. Recognition of eosinophilia may aid in the diagnosis of these conditions and understanding the mechanisms of eosinophilia may provide insights into potential prognostic implications and treatment strategies.

## Introduction

Eosinophilia is defined as an absolute eosinophil count exceeding 0.5 × 10^9^ /L in peripheral blood and is classified as hypereosinophilia if the eosinophil count exceeds 1.5 × 10^9^/L [1]. Tissue eosinophilia, on the other hand, involves an increased eosinophilic infiltrate in the tissue but no defined criteria exist.

Eosinophilia is commonly associated with allergic reactions, parasitic infections, autoimmune diseases, drug reactions, malignancies, and primary eosinophilic disorders [2, 3]. With respect to hematologic malignancies, eosinophilia can be primary (neoplastic) or secondary (reactive).

Neoplastic causes of eosinophilia are often driven by genetic abnormalities and include: myeloid/lymphoid neoplasms with eosinophilia (MLN) that often involve genetic abnormalities such as rearrangements of *PDGFRA*, *PDGFRB*, and *FGFR1*, acute myeloid leukemia (AML) with *CBFB::MYH11* fusion, and chronic myeloid leukemia (CML) driven by the BCR-ABL1 fusion gene [4–6]. Chronic eosinophilic leukemia (CEL) is another primary cause of eosinophilia; however, it has no known specific genetic aberrations though molecular testing is necessary to exclude the aforementioned malignancies [7].

Conversely, reactive eosinophilia can occur in certain hematologic malignancies, typically secondary to cytokines produced by the neoplastic cells or cells within the tumor microenvironment. Hematologic malignancies commonly associated with reactive eosinophilia include Hodgkin lymphoma, mature T-cell lymphomas, lymphocytic variant of hypereosinophilic syndrome (L-HES), and acute lymphoblastic leukemias [8].

Hematopoietic neoplasms with primary or secondary eosinophilia may present as localized head and neck tumors in some cases. Common hematopoietic malignancies that present with eosinophilia in the head and neck region include Hodgkin lymphoma, acute lymphoblastic leukemias, mature T-cell lymphomas, and Langerhans cell histiocytosis [8, 9]. This article aims to provide a comprehensive review of these commonly occurring head and neck hematolymphoid malignancies as well as less common malignancies and explore their association with eosinophilia.

## Classic Hodgkin Lymphoma

**Fig. 1:**
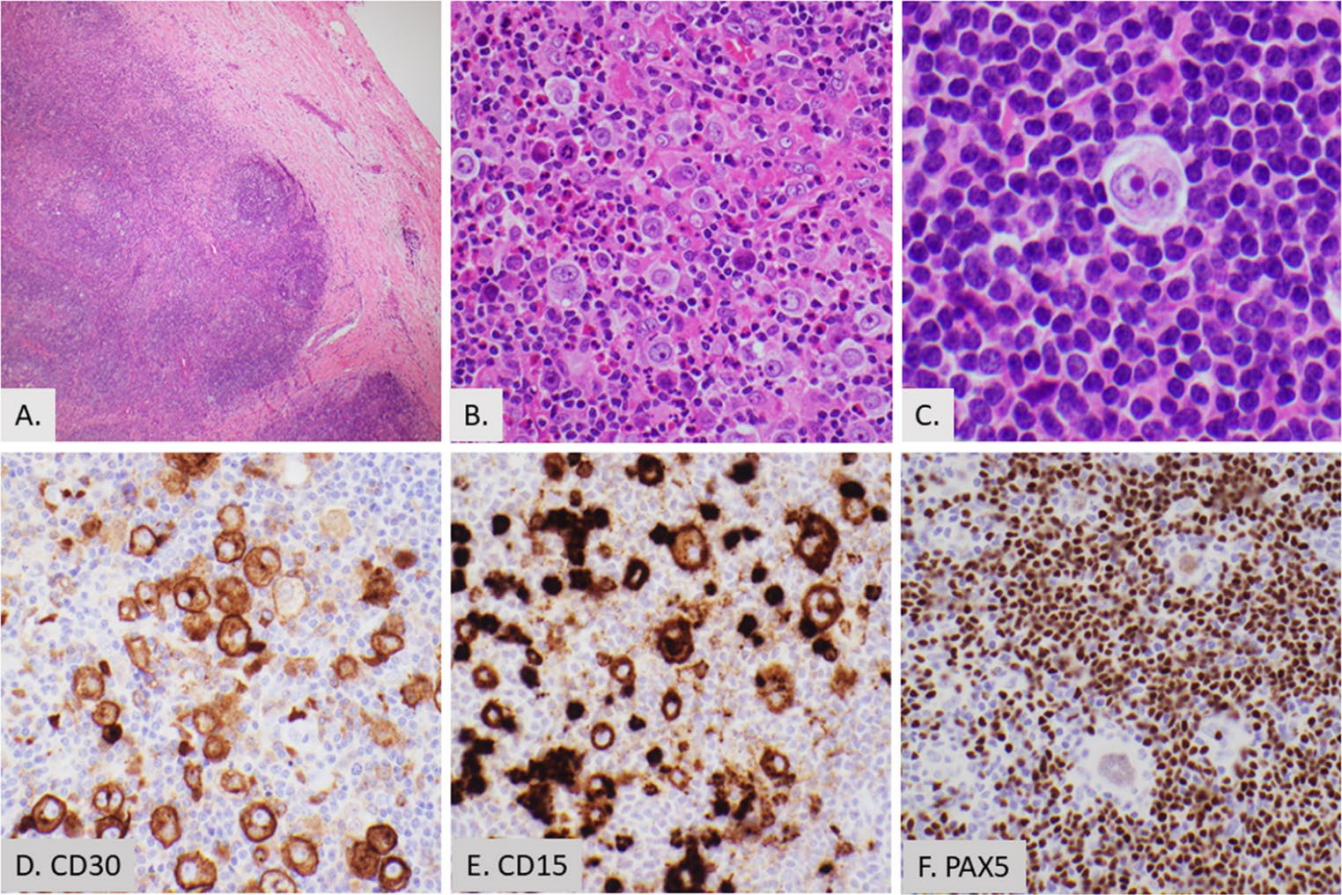
Classic Hodgkin lymphoma with numerous eosinophils. (**A**) Low power showing the architectural features of sclerosis and a nodular proliferation of hematolymphoid cells (2X). (**B**) Hodgkin-Reed-Sternberg (HRS) cells in a polymorphous background of lymphocytes (40X), eosinophils, plasma cells, and histiocytes. **C, D, E,** and **F**. High power of an HRS cell showing positive staining for CD30 (100X) (**D**) and CD15 (40X) (**E**) and weak PAX5 positivity (40X) (**F**)

Classic Hodgkin lymphoma (CHL) predominantly affects supradiaphragmatic lymph nodes, with cervical nodes being the most common site [9]. Although CHL typically arises in lymph nodes, a small subset of cases present as purely extranodal disease [10]. In the head and neck region, extranodal involvement may occur in the tonsils, nasopharynx, thyroid, parotid glands, adenoids, and Waldeyer’s ring [11]. Approximately 15% of CHL cases present with peripheral blood eosinophilia [12, 13]. The neoplastic cells in CHL, known as Reed-Sternberg cells (RS cells) in their multinuclear form and Hodgkin cells in their mononuclear form, are typically a minority of the cells within the tumor. These cells produce a variety of chemokines and cytokines that play critical roles in leukocyte trafficking, contributing to the formation of a complex tumor microenvironment [14]. This microenvironment often contains a mixture of small lymphocytes, plasma cells, histocytes, and eosinophils. [Figure 1]

### Mechanisms of Eosinophilia in CHL

Eosinophilia in CHL is thought to be multifactorial. Interleukin-5 (IL-5) promotes eosinophil growth, differentiation, and activation leading to their increased presence in blood and tissues [15]. The neoplastic cells in CHL (i.e. Reed-Sternberg cells) have been shown to contain the mRNA coding for IL-5, suggesting that direct IL-5 production by these cells contributes to the eosinophilia [16]. Further supporting the role of IL-5 in the recruitment and survival of eosinophils in CHL, eosinophils cultured in serum or supernatant from CHL patients exhibit enhanced survival, which is reversed by the addition of neutralizing anti-IL-5 antibodies.

Eosinophil levels in CHL correlate with elevated CD4 + T-cell counts and an increased T helper 2 (Th2) cytokine profile [17]. Eosinophils secrete immunomodulatory cytokines such as IL-4, IL-6, IL-13, and IL-25 that promote Th2 differentiation [18]. This suggests that IL-5 production in CHL is not solely dependent on neoplastic cells but also involves IL-5 secretion by non-neoplastic Th2 cells.

Thymus and activation-regulated chemokine (TARC) is another contributor to eosinophilia in CHL. TARC, expressed by antigen-presenting cells like dendritic cells recruits Th2 cells by interacting with CCR4. Expression of TARC has been detected in the cytoplasm of RS cells of both nodular sclerosing and the mixed cellularity subtypes of CHL using immunohistochemistry and RNA in situ hybridization [19]. Additionally, elevated serum TARC levels have been observed in CHL patients with higher levels associated with progressive disease [20]. TARC immunohistochemistry may be useful for distinguishing CHL from its mimics. Greater than 90% of CHL cases demonstrated TARC immunohistochemical positivity compared to 6.4% of other mature lymphoid neoplasms with HRS-like cells and none of the reactive lymphadenopathies [21]. 

While IL-5 is necessary for growth, differentiation, and survival of eosinophils, eotaxin is a chemokine critical for eosinophil migration into tissues [22]. Eotaxin has been found to be expressed at a greater rate in tissue involved by CHL, and the amount of eotaxin in tissue has been shown to correlate with the extent of tissue eosinophilia [23]. Studies suggest that increased eotaxin levels in CHL tissue are driven by tumor necrosis factor-alpha (TNF-α) secretion from neoplastic Hodgkin cells, which induces eotaxin expression in fibroblasts [24].

In summary, eosinophilia in CHL involves secretion of eotaxin and TARC secretion by CHL neoplastic cells which in turn leads to recruitment of eosinophils and Th2 cells. Th2 cell and CHL neoplastic cell secretion of IL-5 aids in eosinophil growth and survival. Eosinophils in tissue may further amplify this cycle by secreting Th2 -promoting cytokines such as IL-4, IL-6, IL-13, and IL-25.

### Role of Eosinophilia in CHL Pathogenesis

Eosinophilia is implicated in the pathogenesis and proliferation of CHL neoplastic cells. CD30, a surface glycoprotein of the TNF receptor superfamily, is characteristically expressed in CHL. CD30 signaling is mediated by a signal transducer such as TNF receptor-associated factors (TRAF), which binds to the cytoplasmic tail of CD30, leading to downstream signaling and activation of nuclear factor kappa B (NF-kB) [25]. 

The CD30 ligand (CD30L), is a surface protein that is expressed on bystander cells with higher expression around the CHL neoplastic cells, and it mediates CD30 signaling. CD30L expression has been shown within all subtypes of CHL to varying degrees [26]. Additionally, CD30L mRNA and protein expression has been demonstrated in CHL associated eosinophils [27]. While eosinophils and other bystander cell CD30L expression likely contributes to promoting CHL cellular signaling, it has also been suggested that CD30 overexpression leads to protein self-assembly and TRAF protein recruitment independent of CD30L binding [28].

### Prognostic Implications of Eosinophilia in CHL

In addition to being a useful clue in the diagnosis of Hodgkin lymphoma, tissue eosinophilia in CHL may serve as a potential prognostic indicator. Heavy eosinophil infiltration (defined as > 200 eosinophils per 10 high power fields) has been associated with a significantly worse disease free-survival [29]. Similarly, in patients with CHL, nodular sclerosis subtype, heavy eosinophilia infiltration (defined as > 5% of all cells in at least 5 high power fields) was associated with poorer overall survival and decreased freedom from treatment failure [30]. On the contrary, other publications that used identical definitions of tissue eosinophilia (> 200 eosinophils per high power field [31] and > 5% of cell in 5 high power fields [32]) have found no significant impact of tissue eosinophilia on prognosis or survival. Given these conflicting findings, further research is needed to clarify the relationship between eosinophilia and clinical outcomes in CHL.

## Mature T-cell Neoplasms

**Fig. 2:**
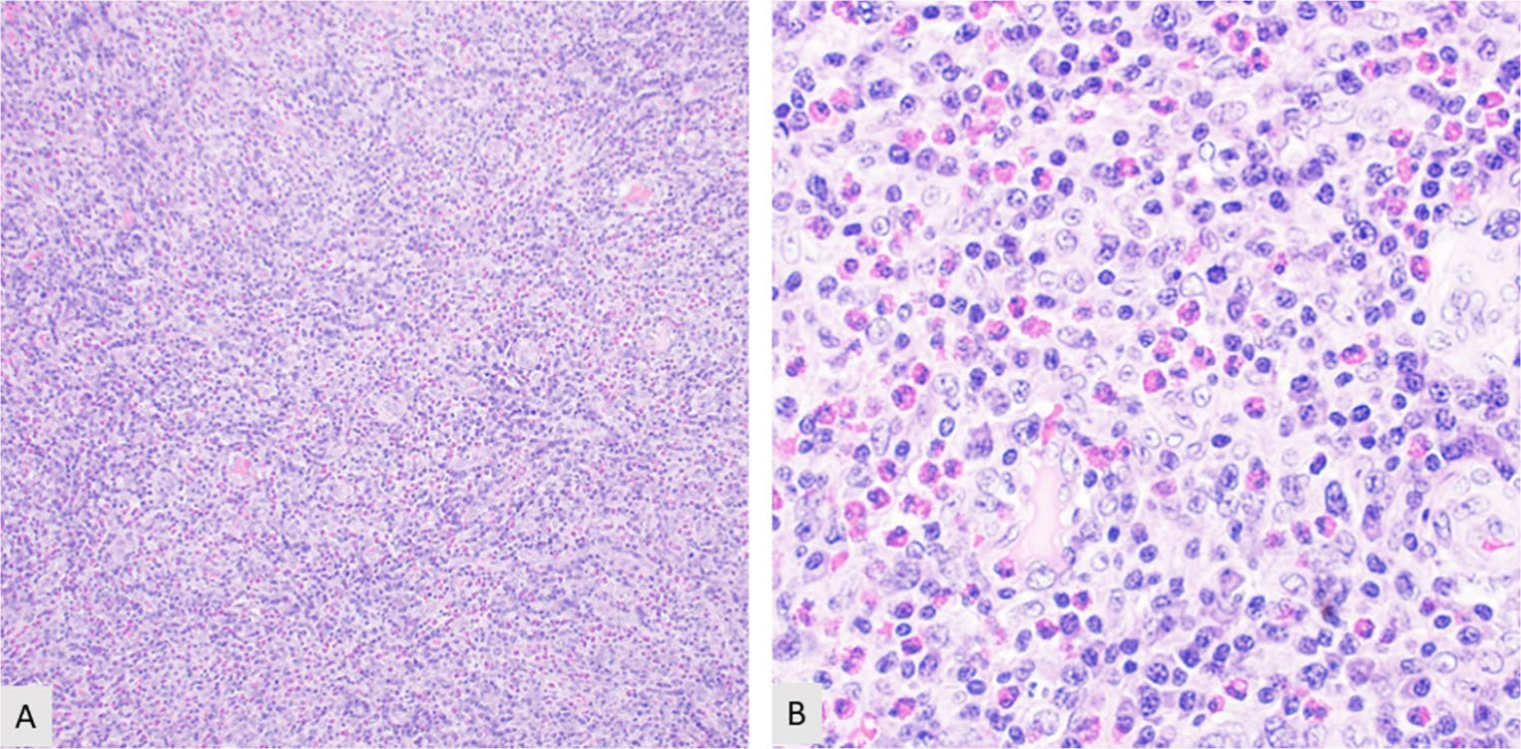
Peripheral T cell lymphoma with numerous eosinophils. (**A**) Effacement of the architecture by small to intermediate atypical lymphocytes (10X). (**B**) Intermediate power of the atypical lymphocytes and numerous eosinophils (20X)

**Fig. 3:**
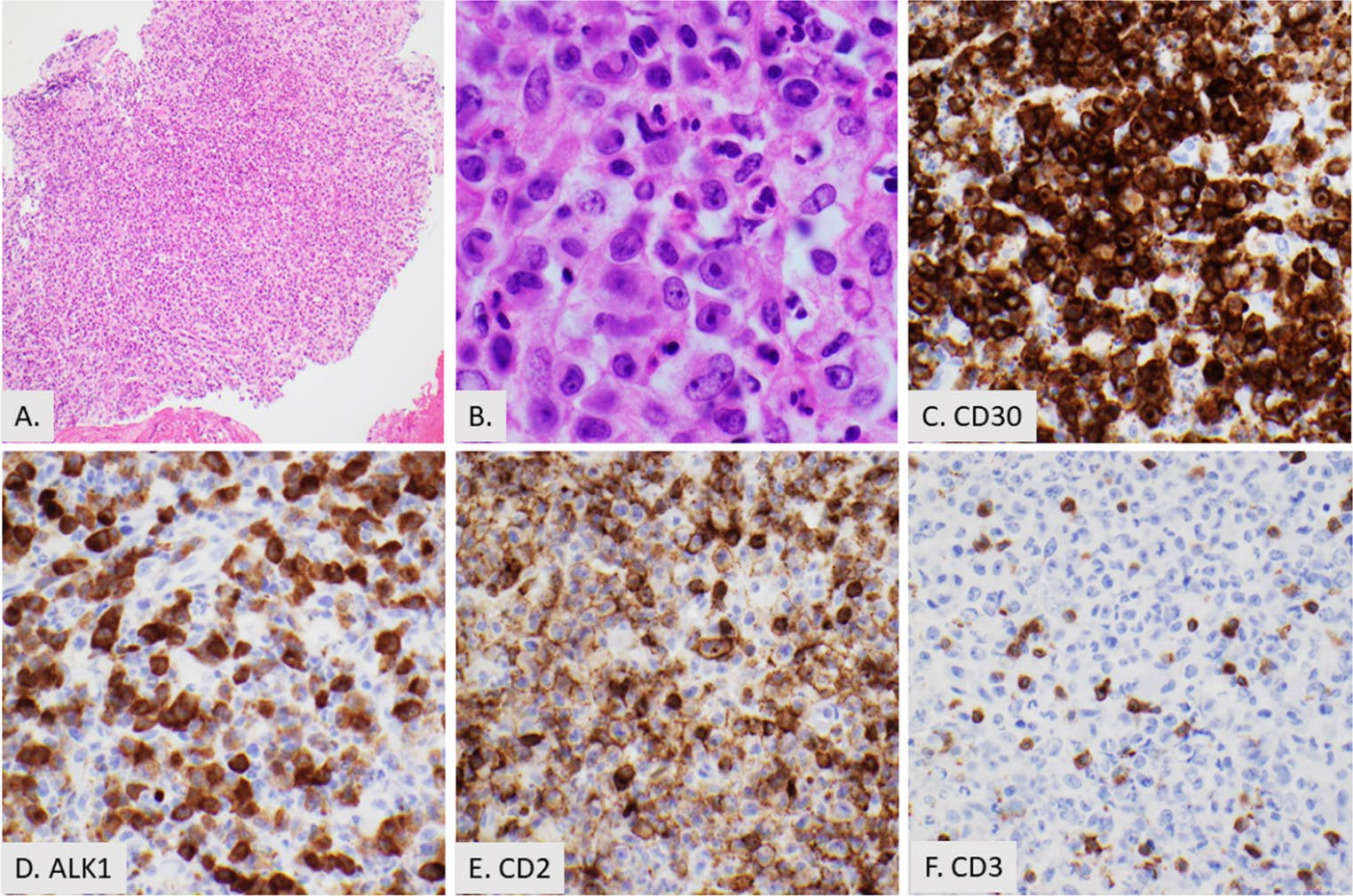
Anaplastic large cell lymphoma with numerous eosinophils in the upper airway. (**A**) Low power image of effaced lymphoid tissue (2X). (**B**) High power showing large atypical cells with neutrophils and numerous eosinophils (40X). **C, D, E**, and **F**. The tumor cells show uniform, strong expression of CD30 (20X) (**C**), are positive for ALK1 (20X) (**D**) and CD2 (20X) (**F**), and negative for CD3 (20X) (**F**)

Mature T-cell neoplasms (MTCN) encompass a diverse group of diseases accountable for roughly 15% of non-Hodgkin lymphomas. Peripheral T-cell lymphoma, not otherwise specified (PTCL-NOS), [Fig. 2] is the most common subtype and constitutes roughly a quarter of all MTCNs [33]. The angioimmunoblastic type of nodal T-follicular helper cell lymphoma (NTFHL-AI), is the second most common MTCN representing approximately 20% of cases [34]. Adult T-cell lymphoma/leukemia (ATLL) is a T-cell lymphoma associated with human T-cell lymphotropic virus type 1 (HTLV-1). ATLL predominantly affects individuals in regions where HTLV-1 is endemic, accounting for approximately 25% of MTCNs in Japan, compared to 1–2% in Europe and North America [35]. The most common types of mature T cell neoplasms typically present with generalized lymphadenopathy but can occasionally present localized to extranodal sites within the head and neck such as Waldeyer’s ring [36, 37]. Other types of mature T cell lymphomas have more specific presentations such as CD30 positive T-cell LPD (commonly seen in the skin) and anaplastic large cell lymphoma [Fig. 3] that may be ALK-positive (commonly in the lymph node) or ALK-negative (present in either the skin or lymph node) [38]. 

### Mechanism of Eosinophilia in Mature T-cell Neoplasms

Peripheral blood and tissue eosinophilia may be present in PTCL-NOS, NTFHL-AI, CD30-LPD, and ATLL. This eosinophilia results from complex interactions between malignant T-cells and the tumor microenvironment. Immunohistochemical analysis of TARC and IL-5 has demonstrated higher expression in PTCL-NOS and NTFHL-AI cases with eosinophilia compared to those without. In these cases, TARC expression is primarily identified in non-lymphoid cells with dendritic morphology, whereas IL-5 expression is more pronounced in neoplastic lymphocytes. Moreover, cases with elevated TARC expression often show concurrent high IL-5 expression [39]. In ATLL, high serum IL-5 levels are present in a subset of cases, correlating significantly with eosinophilia [40]. The neoplastic T-cells in ATLL exhibit increased TARC production compared to background, nonneoplastic lymphocytes [41]. Overall, these findings suggest a direct relationship between TARC, IL-5, and eosinophilia in PTCL-NOS NTFHL-AI, and ATLL. Interestingly, TARC expression is typically absent in reactive lymphadenopathies and may offer a unique marker for differentiating these neoplasms from reactive mimics [21].

### The Role of Epstein Barr Virus in Eosinophilia

Epstein Barr virus (EBV) is another potential contributor to eosinophilia in T cell neoplasms particularly in PTCL-NOS and NTFHL-AI. EBV is detected in approximately 21% of PTCL-NOS cases and 74–91% of NTFHL-AI cases [42]. EBV can infect both B-cells and T-cells, often producing large immunoblast-like cells sometimes with multiple nuclei and prominent nucleoli, resembling Reed-Sternberg cells [43]. IL-5 is typically expressed by T-cells; however, expression may be induced in B-cells after EBV infection. In vitro studies have shown that EBV transformed B-cells produced IL-5 encoding mRNA with corresponding IL-5 detected on western blot. Moreover, the culture supernatant of the EBV transformed B-cells supported the selective growth of eosinophil colonies in a semi-solid culture [44]. These findings suggest that immune dysregulation caused by EBV may contribute to creating a pro-eosinophilic environment.

### Prognostic Implications of Eosinophilia in Mature T-cell Neoplasms

Little is known about the prognostic significance of eosinophilia in most mature T cell neoplasms. However, eosinophilia has been shown to be a poor prognostic factor in ATLL. Cases with a high IL-5, defined as greater than 1.7 pg/ml, were found to have a significantly lower overall survival of 7.3 months compared to 13.3 months in those without high serum IL-5 [40]. Moreover, blood eosinophilia has also been shown to be an independent unfavorable prognostic factor in patients with ATLL [45]. Further studies are necessary to determine the unfavorable prognostic factor of eosinophilia seen in ATLL is also seen in PTCL-NOS and NTFHL-AI.

## Extranodal NK/T Cell Lymphomas (ENKTL)

**Fig. 4:**
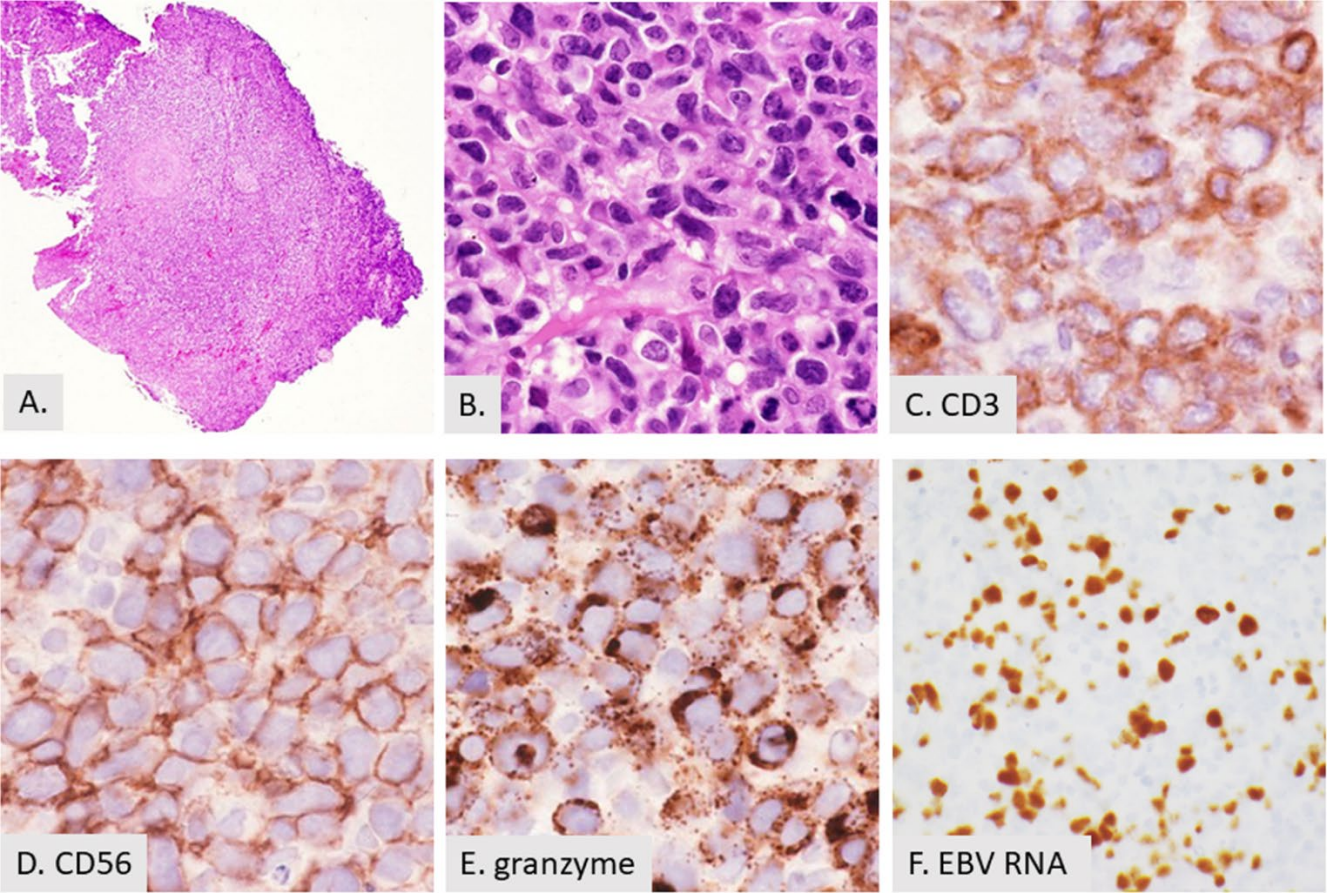
NK/T cell lymphoma of the hard palate. **A**. Low power showing architectural effacement (2X). **B, C, D, E,** and **F**. High power images showing large atypical cells with abundant cytoplasm (40X) (**B**), which show cytoplasmic CD3 expression (40X) (**C**), are positive for CD56 (40X) (**D**), granzyme (40X) (**E**), and EBV RNA (40X) (**F**). The cells are also positive for TIA1 and CD2 and are negative for CD5 (not shown)

Extranodal NK/T cell lymphoma (ENKTL) typically presents in the head and neck with symptoms of nasal obstruction, epistaxis and necrotizing lesions of the nose or hard palate and rarely peripheral eosinophilia is observed [46]. Diagnosis can be challenging, often requiring multiple biopsies due to the extensive inflammation and necrosis. ENKTL is strongly associated with EBV infection. [Figure 4] Histologically, ENKTL displays a polymorphous infiltrate comprising neoplastic cells, small lymphocytes, plasma cells, histiocytes and eosinophils [46–48]. The neoplastic cells are variable in size, but many are medium-sized with clear cytoplasm, irregular nuclear contours, inconspicuous nucleoli, and granular chromatin [38]. The growth pattern is often angiocentric and there is also extensive necrosis. Tumors in the nasal cavity can show a pseudoepitheliomatous hyperplasia in the overlying the squamous mucosa [1]. The neoplastic cells commonly have an NK-cell immunophenotype but can have a T-cell lineage in roughly 40% of cases [49]. The pathogenesis and prognostic significance of eosinophilia in ENKTL remain unknown.

## T-cell Acute Lymphoblastic Leukemia/lymphoma

T-cell acute lymphoblastic leukemia/lymphoblastic lymphoma (T-ALL/LBL) is an aggressive hematologic malignancy characterized by the proliferation of T-lymphoblasts. T-ALL/LBL accounts for between 15% and 25% of all acute lymphoblastic leukemia (ALL) cases in both children and adults. It most commonly presents in adolescents and young adults as a rapidly expanding mediastinal mass originating from the thymus [50]. In some cases, the rapid expansion of the mediastinal mass can compress the superior vena cava, resulting in superior vena cava syndrome or it can compress the trachea, leading to respiratory distress [51]. Pleural and/or pericardial effusions may also be observed in certain patients [52]. 

### Mechanism of Eosinophilia in T-ALL/LBL

Eosinophilia is present in some cases of T-ALL/LBL and is believed to be driven by cytokine production from the neoplastic cells, promoting eosinophil growth and survival. Specific genetic aberrations have been implicated in the development of eosinophilia. One such example is the t(5;7) (q31;q21)/*CDK6::IL3* rearrangement, which has been associated with increased IL-3 expression and subsequent eosinophilia. This chromosomal translocation relocates the *IL3* gene near the CDK6 enhancer, resulting in IL-3 overexpression [53]. IL-3 binds to its receptor, IL-3 receptor subunit alpha (IL-3Rα), on eosinophils, triggering intracellular signaling pathways that enhance protein production and prolong cell survival, ultimately leading to eosinophilia [54]. Currently, there are no reports in the literature regarding the prognostic significance of eosinophilia in T-ALL/LBL.

## Primary Mucosal CD30-positive T-cell Lymphoproliferative Disorder

**Fig. 5:**
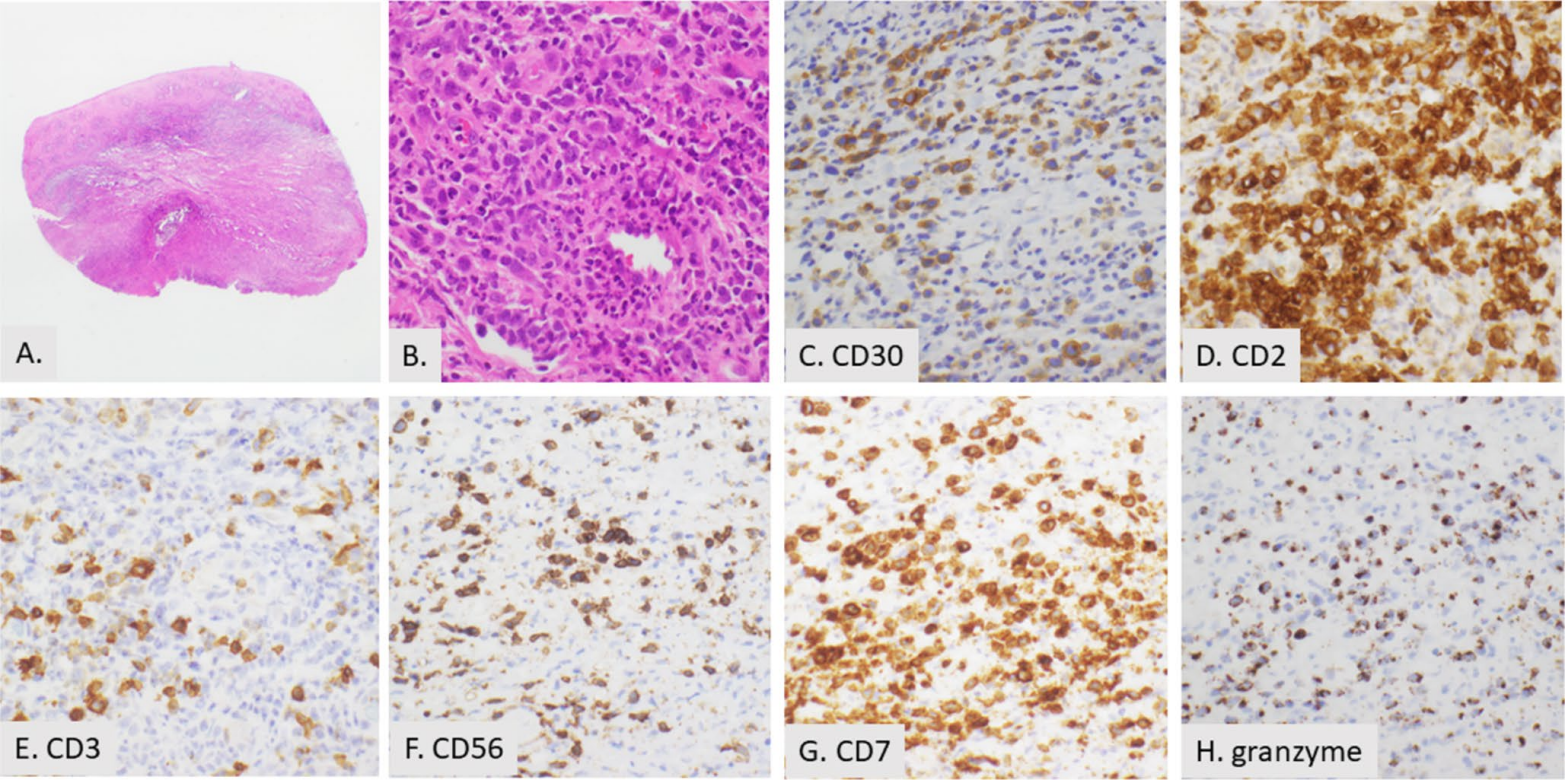
CD30 mucocutaneous lesion in the mouth. (**A**) Low power image of oral mucosa with necrosis and an atypical large cell infiltrate (2X). (**B**) Large cells are scattered in the tissue and perivascular (20X). **C, D, E, F, G**, and **H**. The majority of large atypical cells are positive for CD30 (20X) (**C**), CD2 (20X) (**D**), CD7 (20X) (**G**), and granzyme (20X) (**H**) and subset positive for CD3 (20X) (**E**) and CD56 (20X) (**F**). The cells were negative for CD5, TCRBF1, TCR delta, CD8, CD4, CD56, perforin EBV and ALK (not shown). The lesion was indolent and resolved

Primary mucosal CD30-positive T-cell lymphoproliferative disorder (CD30 + LPD) [Fig. 5] in the head and neck region represents a clinicopathologic spectrum of lymphoproliferative lesions characterized by the expression of CD30 on atypical lymphoid cells [55]. CD30 + LPD can manifest in head and neck mucosal sites, most commonly in the oral cavity but can also occur in the nasopharynx and other parts of the upper aerodigestive tract [56, 57]. These lesions have similar morphology to primary cutaneous CD30-positive T cell lymphoproliferative disorder and may be the mucosal equivalent. These lesions often have tissue eosinophilia and show histologic overlap with traumatic ulcerative granuloma with stromal eosinophilia (TUGSE). Interestingly, many cases of TUGSE have atypical CD30 positive cells and may have T-cell monoclonality, which suggests that TUGSE may exist on a spectrum with CD30 + LPD [58, 59]. Cases of CD30 + LPD have an indolent course and may spontaneously regress with recurrence after treatment being uncommon [55, 56, 59]. Occasional cases have rearrangements of *DUSP22* on 6p25.3 [9]. There is currently no research into the mechanism of eosinophilia in specifically CD30 + LPD; however, similar to other entities discussed in this article, it likely involves the secretion of pro-eosinophilic cytokines by bystander Th2 T-cells and potentially by the neoplastic cells. Due to the indolent nature of the disease and its excellent prognosis, proper diagnosis of the disease is critical. Recognition of eosinophilia may provide an important diagnostic reminder to include this disease in the differential diagnosis.

## Langerhans Cell Histiocytosis

**Fig. 6:**
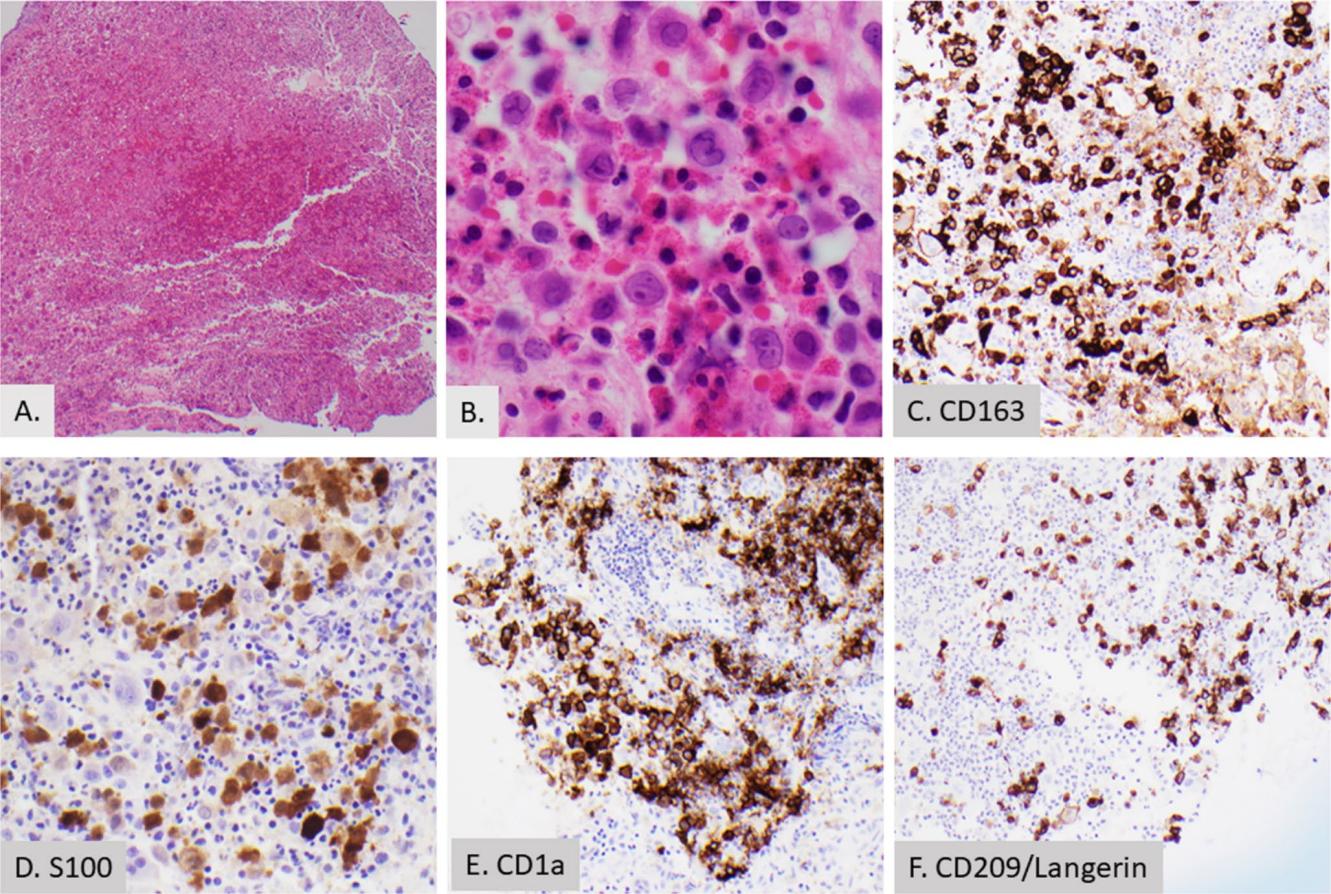
Langerhans cell histiocytosis in an 11 year old with an ear mass. (**A**) Low power showing large, atypical cells with numerous eosinophils (2X). (**B**) High power image showing large cells with indentations and abundant cytoplasm with background eosinophils (40X). **C, D, E**, and **F**. The neoplastic cells are positive for CD163 (20X) (**C**), S100 (20X) (**D**), and CD1a (20X) (**E**) and a subset of cells is positive for Langerin (20X) (**F**).Factor XIII and BRAF are negative (not shown)

Langerhans cell histiocytosis (LCH) [Fig. 6] is a rare neoplasm characterized by the clonal proliferation of myeloid dendritic cells which express CD1a and CD207 (langerin). LCH most commonly occurs in children with a median age of 3.5 years and can involve a single organ system (single-system LCH) or multiple systems (multisystem LCH) [60]. LCH may manifest as head and neck lesions which most commonly involve the skull, followed by the cervical lymph nodes [61]. Tissue eosinophilia is a notable histopathologic feature in many cases of LCH; although, peripheral eosinophilia is not commonly reported.

### Mechanism of Eosinophilia in LCH

Tissue eosinophilia in LCH is largely attributed to local production of cytokines and chemokines. The non-neoplastic background T-cells in LCH cases produce many cytokines including IL-2, IL-3, IL-4, IL-5, granulocyte-macrophage colony-stimulating factor (GM-CSF), and TNF-α. In addition, LCH neoplastic cells produce IL-1α, IFN-γ, and GM-CSF. Eosinophils, if present, also contribute to the complex chemokine/cytokine local environment with notable production of IL-5, IL-7, IL-10, IFN-γ, and GM-CSF [61]. As discussed previously, IL-3 and IL-5 play a crucial role in the growth, differentiation, and activation of eosinophilia. While not as crucial as IL3 and IL5, GM-CSF, IFN-γ, and TNF-α also interact with eosinophils contributing to eosinophil activation and stimulation of effector function [62–64]. 

## Myeloid/lymphoid Neoplasms with Eosinophilia

Myeloid/lymphoid neoplasms with eosinophilia (MLN) and tyrosine kinase (TK) gene fusions are frequently associated with clonal eosinophilia and may present as myeloproliferative neoplasms (MPNs), myelodysplastic/myeloproliferative neoplasms (MDS/MPN), AMLs, and/or acute lymphoblastic leukemias/lymphomas [65]. MLN typically manifests as a diffuse process but may rarely present as a localized head and neck mass [7]. The World Health Organization (WHO) 5th edition classification defines several distinct MLN entities based on the specific gene rearrangements including: MLN with *PDGFRA* rearrangement, MLN with *PDGFRB* rearrangement, MLN with *FGFR1* rearrangements, MLN with *JAK2* rearrangements, MLN with *FLT3* rearrangements, and MLN with *ETV6::ABL1* fusion. [1]

TK gene fusions activate the TK signaling pathway activation through two primary mechanisms: (1) Disruption of the Inhibitory Domain and (2) Formation of Multimers. Most TK proteins possess an inhibitory domain that maintains them in an inactive state. TK gene fusions often disrupt this domain leading to the protein to be maintained in the active conformation. Alternatively, the TK partner gene may induce the formation of multimers, which also results in constitutive activation of the TK pathway [66]. In both mechanisms, unregulated TK mediated cellular signaling promotes cell survival and cell proliferation. In MLN, these gene rearrangements are thought to originate in pluripotent hemopoietic progenitor cells that can differentiate into multiple lineages, contributing to the variable clinical presentations and clonal eosinophilia in these neoplasms [67]. 

Importantly, because MLN is a primary cause of eosinophilia involving TK gene fusions, prognosis may be improved with TK-inhibitor (TKI) therapy in some cases. MLN with *PDGFRA* rearrangements have an exceptionally good prognosis due to imatinib therapy, with the majority of patients achieving complete remission [68, 69]. In rare cases of MLN with *PDGFRA* rearrangements that develop resistance to imatinib, alternative TKI therapies have shown effectiveness [70, 71]. Similar to MLN with *PDGFRA*, MLN with *PDGFRB* responds well to imatinib therapy with a 10-year overall survival rate of around 90% [72]. MLN with *FGFR1* rearrangements follows a more aggressive course compared to the previous two entities, with a high incidence of myeloid or T-cell phenotype blast transformation [4]. The FGFR1 rearranged cases have shown resistance or limited response to TKI therapy including imatinib and ponatinib, requiring most patients to undergo allogenic hemopoietic cell transplantation (HCT) [73, 74]. There are some TKI therapies that have shown promise in these cases including futibatinib, a small molecule inhibitor of FGFR1-4, which has shown durable remission in one case report [75]. Additionally, there is an ongoing clinical trial evaluating the TKI pemigatinib in patients with MLN with *FGFR1* rearrangements [76]. MLN with *JAK2* rearrangements and MLN with *FLT3* rearrangements have shown poor or only temporary response to TKI therapy, making allogenic HCT the typical treatment option [77–79]. Overall, the susceptibility of certain MLN to TKIs highlights the importance of understanding the underlying mechanisms of clonal eosinophilia. Continued advancement in targeted therapies has the potential to significantly improve patient outcomes in these rare and often aggressive neoplasms.

## Conclusion

Eosinophilia is a notable feature in various hematologic malignancies affecting the head and neck. The presence of eosinophilia, whether primary or secondary, offers valuable diagnostic insights and may carry prognostic significance. Primary eosinophilia is often driven by genetic abnormalities, such as tyrosine kinase gene fusions in MLNs. In contrast, secondary eosinophilia arises from cytokine and chemokine production by the neoplastic cells or the tumor microenvironment. Key mediators involved in eosinophil growth, activation, proliferation, and tissue migration include the chemokines and cytokines, IL-3, IL-5, eotaxin, and TARC. A deeper understanding of the mechanisms driving eosinophilia can aid in the diagnosis, prognostication, and management of these malignancies. Microenvironments in these lymphoproliferative processes and how they impact lymphomagenesis are not well understood. The development of drugs targeting the cellular and cytokine milieu may offer new therapeutic options to help mitigate symptoms and improve outcomes for patients in the future [48]. 

## Data Availability

No datasets were generated or analysed during the current study.

## Declarations

### Disclaimer

The views expressed herein are those of the authors and do not necessarily reflect the official policy or position of Walter Reed National Military Medical Center, Fort Belvoir Community Hospital, the U.S. Army Medical Department, the U.S. Army Office of the Surgeon General, the Department of the Air Force, the Department of the Army, Department of Defense, the Uniformed Services University of the Health Sciences, or any other agency of the U.S. Government. The identification of specific products or scientific instrumentation is considered an integral part of the scientific endeavor and does not constitute endorsement or implied endorsement on the part of the authors, DoD, or any component agency.

### Statistical Declaration

No statistical analysis was performed for this manuscript.
